# Ring-Opening Polymerization of ε-Caprolactone and Styrene Oxide–CO_2_ Coupling Reactions Catalyzed by Chelated Dehydroacetic Acid–Imine Aluminum Complexes

**DOI:** 10.3390/molecules27010164

**Published:** 2021-12-28

**Authors:** Ting-Yen Wang, Yu-Chia Su, Bao-Tsan Ko, Yu Hsu, Yu-Fang Zeng, Ching-Han Hu, Amitabha Datta, Jui-Hsien Huang

**Affiliations:** 1 Department of Chemistry, National Changhua University of Education, Changhua 500, Taiwan; tingyen0514@gmail.com (T.-Y.W.); s0625044@gm.ncue.edu.tw (Y.H.); s411070@nhes.edu.tw (Y.-F.Z.); chingkth@cc.ncue.edu.tw (C.-H.H.); amiticju007@yahoo.co.in (A.D.); 2 Department of Chemistry, National Chung-Hsing University, Taichung 402, Taiwan; jack30148@yahoo.com.tw (Y.-C.S.); btko@dragon.nchu.edu.tw (B.-T.K.)

**Keywords:** aluminum, chelated dehydroacetic acid–imine ligands, ring-opening polymerization, styrene oxide-CO_2_ coupling reactions, ε-caprolactone

## Abstract

A series of chelated dehydroacetic acid–imine-based ligands **L^1^H**~**L^4^H** was synthesized by reacting dehydroacetic acid with 2-t-butylaniline, (*S*)-1-phenyl-ethylamine, 4-methoxylbenzylamine, and 2-(aminoethyl)pyridine, respectively, in moderate yields. Ligands **L^1^H**~**L^4^H** reacted with AlMe_3_ in toluene to afford corresponding compounds **AlMe_2_L^1^** (**1**), **AlMe_2_L^2^** (**2**), **AlMe_2_L^3^** (**3**), and **AlMe_2_L^4^** (**4**). All the ligands and aluminum compounds were characterized by IR spectra, ^1^H and ^13^C NMR spectroscopy. Additionally, the ligands **L^1^H**~**L^4^H** and corresponding aluminum derivatives **1**, **3**, and **4** were characterized by single-crystal X-ray diffractometry. The catalytic activities using these aluminum compounds as catalysts for the ε-caprolactone ring-opening polymerization (ROP) and styrene oxide-CO_2_ coupling reactions were studied. The results show that increases in the reaction temperature and selective solvent intensify the conversions of ε-caprolactone to polycaprolactone. Regarding the coupling reactions of styrene oxide and CO_2_, the conversion rate is over 90% for a period of 12 h at 90 °C. This strategy dispenses the origination of cyclic styrene carbonates, which is an appealing concern because of the transformation of CO_2_ into an inexpensive, renewable and easy excess carbon feedstock.

## 1. Introduction

**Scheme 1:**
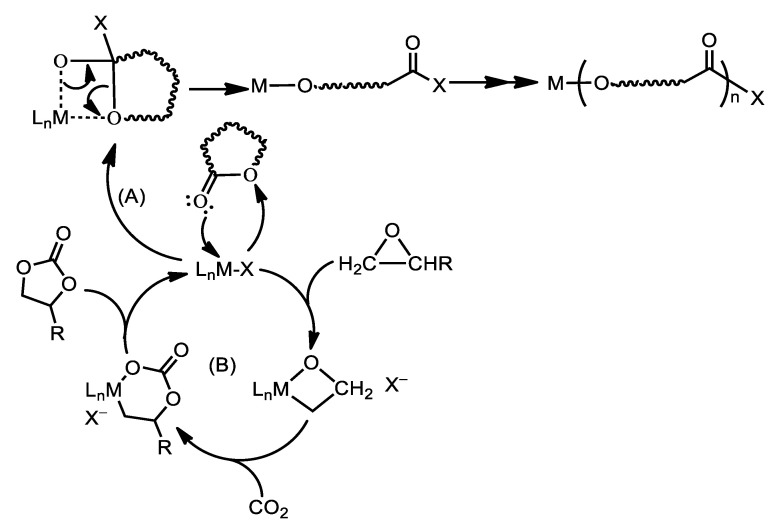
(**A**) The catalytic reactions of ring-opening polymerization of a cyclic ester; (**B**) the catalytic cycles of epoxide–CO_2_ coupling.

Many issues regarding the environmental situation have increased the attention being paid to it among the scientific community. Among these issues, pollution is an important problem nowadays due to the extent of human activities and products, and caused the deaths of over 9 million people in 2015 [1]. Different types of pollution, including air pollution, plastic pollution, metal waste pollution, etc., have attracted a lot of attention. Many chemists are also trying to address these issues, and many strategies have been developed as solutions to solve these problems. Among these issues, we are particularly interested in synthesizing poly ε-caprolactone and cyclic carbonate. Poly ε-caprolactone (PCL), a bio-degradable and bio-compatible polymer, has been used in many applications, such as drug delivery systems, tissue engineering, etc., and is considered to be a substitute for fossil-based plastics [2,3,4]. Ring-opening polymerization of ε-caprolactone using metal catalysts is considering to be an efficient method. Many metal catalysts have been studied and developed for catalyzing ε-caprolactone ring-opening polymerization [5,6,7]. Cyclic carbonates have low toxicity and low vapor pressure, and are used as precursors for polymers, acyclic carbonates, reagents, solvents, diluents, etc. The methods for synthesizing cyclic carbonates include epoxide–CO_2_ or urea–polyol coupling reactions [8,9,10,11,12]. Catalysts used for the coupling reactions have been reviewed in many papers in recent years [13,14,15,16,17,18]. With respect to the homogeneous catalysts for the ring-opening polymerization of ε-caprolactone [19,20,21,22] and epoxide–CO_2_ coupling reactions [23,24], common features of these catalysts include both of them being classified as Lewis acid metal complexes bearing varieties of organic ligands. Their reaction mechanisms are shown in Scheme 1, where Scheme 1A presents the ring-opening polymerization of a cyclic ester and Scheme 1B presents the catalytic cycles of epoxide–CO_2_ coupling reactions.

We have been involved in organometallic chemistry, which includes the synthesis, characterization and reactivity study of main and transition metal complexes and their catalytic activity toward cyclic esters ring-opening polymerization, for the past twenty years [25,26,27]. It is known that even minor changes of the ligands on metal complexes can have large effects on their catalytic activities. Here, we have selected bi- or tri-dentate dehydroacetic acid–imine ligands due to their excellent chelating capacity in modern coordination chemistry, especially for group 3A metals. These types of ligands are considered to be distinctly interesting and desirable materials owing to their unusual characteristics, which notably include resource-rich structures, easy synthetic procedures, and diverse chemical applications. Herein, we selected these ligands to determine their catalytic activity with respect to CO_2_ coupling reactions and ring-opening polymerization. Thus, we report the synthesis and characterization of a series of aluminum compounds containing bi- or tri-dentate dehydroacetic acid–imine ligands and their corresponding application in epoxide–CO_2_ coupling reactions and the ring-opening polymerization of ε-caprolactone.

## 2. Results and Discussion

### 2.1. Synthesis and Characterization of ***L^1^H**~**L^4^H*** and Compounds ***1**–**4***

**Scheme 2:**
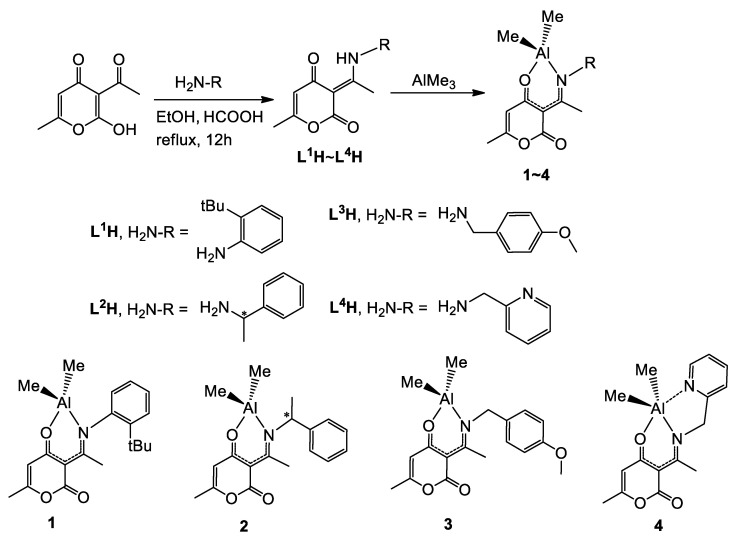
Synthesis of the ligands, **L^1^H**~**L^4^H**, and compounds **1**~**4**.

A series of bidentate and tridentate dehydroacetic acid–imine ligands were obtained from the reactions of dehydroacetic acid and amines in ethanol via imine condensation [28] with a small amount of formic acid as catalyst. Refluxing dehydroacetic acid with one equivalent of primary amine such as 2-t-butylaniline, (*S*)-1-phenyl-ethylamine, 4-methoxylbenzylamine, and 2-(aminoethyl)pyridine in ethanol generated **L^1^H**~**L^4^H**, respectively (Scheme 2). All the ligands were purified via recrystallization and were obtained in moderate yields. The NMR spectra of **L^1^H**~**L^4^H** are relatively similar, all showing characteristic methine fragments of the dehydroacetic acid rings at δ 5.75~5.63 ppm for the ^1^H NMR signals and ca. δ 107 ppm for the ^13^C{^1^H} NMR signals. The ligands **L^1^H** and **L^2^H** have bulkier substituents adjacent to the imine nitrogen atoms and provide the metal center better protection after insertion of the ligands. In contrast, ligands **L^3^H** and **L^4^H** have benzyl or picolyl fragments on the imine nitrogen atoms, respectively resulting in less steric protection or the provision of additional coordinating sites to the metal atoms of the corresponding metal complexes.

**Figure 1:**
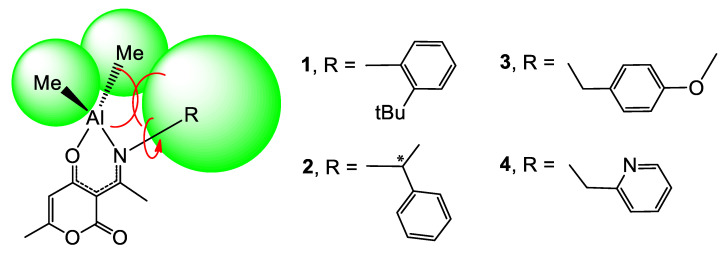
A schematic drawing of the aluminum compounds **1**–**4** showing the steric hindrance of the Al-Me groups with the imine substituents, * denoted chiral center.

Reactions of one equivalent of AlMe_3_ with **L^1^H**~**L^4^H** in toluene at room temperature give corresponding aluminum compounds **AlMe_2_L^1^** (**1**), **AlMe_2_L^2^** (**2**), **AlMe_2_L^3^**(**3**), and **AlMe_2_L^4^** (**4**), respectively, in 65~86% yields. The methine fragments of the pyrone rings of compounds **1**–**4** show one singlet at ca. δ 5.42~5.69 ppm for the ^1^H NMR resonances and one peak at ca. δ 105.0~106.0 ppm for the ^13^C{^1^H} NMR spectra. Interestingly, we noticed that the methyl groups of the AlMe_2_ fragment appear as two singlets for compounds **1** and **2**, whereas only one singlet is observed for compounds **3** and **4**. Presumably, the steric hindrance of substituted imine fragments plays an important role for the stereo-geometries of compounds **1**~**4**. The varible temperature ^1^H NMR spectra of compounds **1**~**4** were measured in the range of 243 K to 333 K in *d*^8^-toluene using a 300 MHz NMR spectrometer. The results indicate that the C-N bond rotation energy barrier is correlated with the R groups of the imine substituents, as shown in Figure 1, where the larger R group is responsible for a higher rotation energy barrier.

Consequently, compounds **1** and **2** have large R groups along with higher C-N bond rotation energy barrier emerging the two methyl NMR signals of AlMe_2_ at room temperature. After increasing the temperature to 60 °C, compound **2** reveals two well-separated AlMe_2_ resonances, whereas **1** exhibits only one signal at ca. 55 °C. The C-N bond rotation energy barriers are estimated at ca. 71.2 and 68.3 kJ/mol for compounds **1** and **2**, respectively [29,30]. On the other hand, the lower C-N steric congestions of compounds **3** and **4** display only one methyl resonance even at −30 °C, reflecting the low C-N rotation energy barrier.

The FTIR spectra of all of the ligands and the corresponding metal complexes were performed in the region 4000–400 cm^−1^. The free ligands show three strong bands in the 1710–1740, 1680–1690 and 1570–1590 cm^−1^ regions, which were attributed to the ν(C=O) of lactone, the ν(C=O) of the pyran ring, and the ν(C=N) stretching frequencies, respectively. These data support the imine ketone resonance form of the ligand. The intense peaks at 1150–1160 cm^−1^ were attributed to the stretching vibration of ν(C-O-C) of the lactone ring, whereas the sharp band at 3060 cm^−1^ corresponds to the aromatic ν(=CH) stretching vibration. A weak broad band emerges in the 2700–3000 cm^−1^ regions, and might be due to the presence of intramolecular hydrogen bonding in **L^2^H**, correlated with its high boiling point of 130 °C. Conversely, no such significant broad bands are perceived in the other three ligands. In all complexes, the ν(C=O) bands increased to 1750 cm^−1^, and ν(C=N) decreased to 1560 cm^−1^, which were attributed to the fact that the ligands were coordinated to the Al metal.

### 2.2. Molecular Structures of Compounds ***L^1^H**~**L^4^H***, ***1***, ***3***, and ***4***

**Scheme 3:**
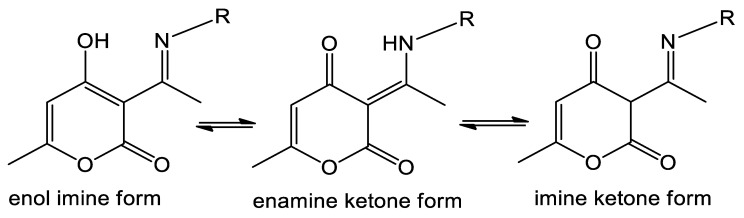
The tautomeric forms of dehydroacetic acid–imine compounds.

Single crystals of **L^1^H**~**L^4^H** suitable for X-ray diffraction were grown from saturated heptane at −20 °C. The molecular structures of **L^1^H**~**L^4^H** are shown in Figures S1–S4. A summary of the data collection process, along with the refinement parameters and selective bond lengths and angles, is provided in Tables S1 and S2, respectively. It is known that dehydroacetic acid and its related imine derivatives exist in enol imine, enamine ketone, and imine ketone tautomeric forms [31,32,33], as shown in Scheme 3.

**Scheme 4:**
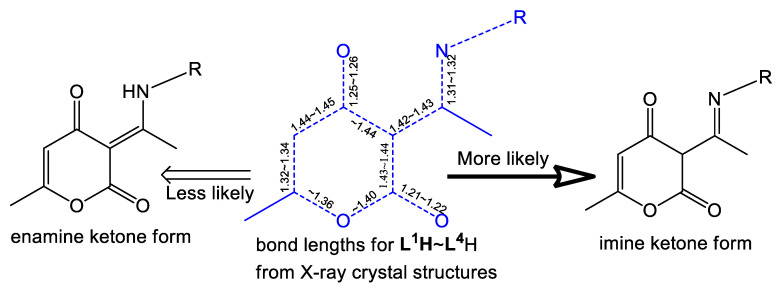
A summarization of bond lengths for **L^1^H**~**L^4^H**, and their possible resonance forms.

The corresponding bond lengths for **L^1^H**~**L^4^H**, are relatively similar to those described in the literature [34,35,36,37]. However, after analyzing the corresponding bond lengths of **L^1^H**~**L^4^H**, we conclude that these ligands in solid state most likely belong to the imine ketone form with a small percentage of enamine ketone (Scheme 4).

**Figure 2:**
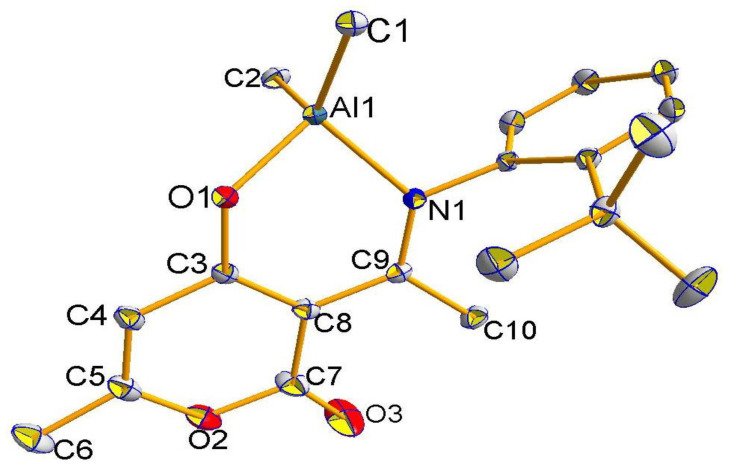
The molecular geometry of compound **1**. Thermal ellipsoids are drawn at the 30% probability level. The hydrogen atoms are omitted for clarity.

**Figure 3:**
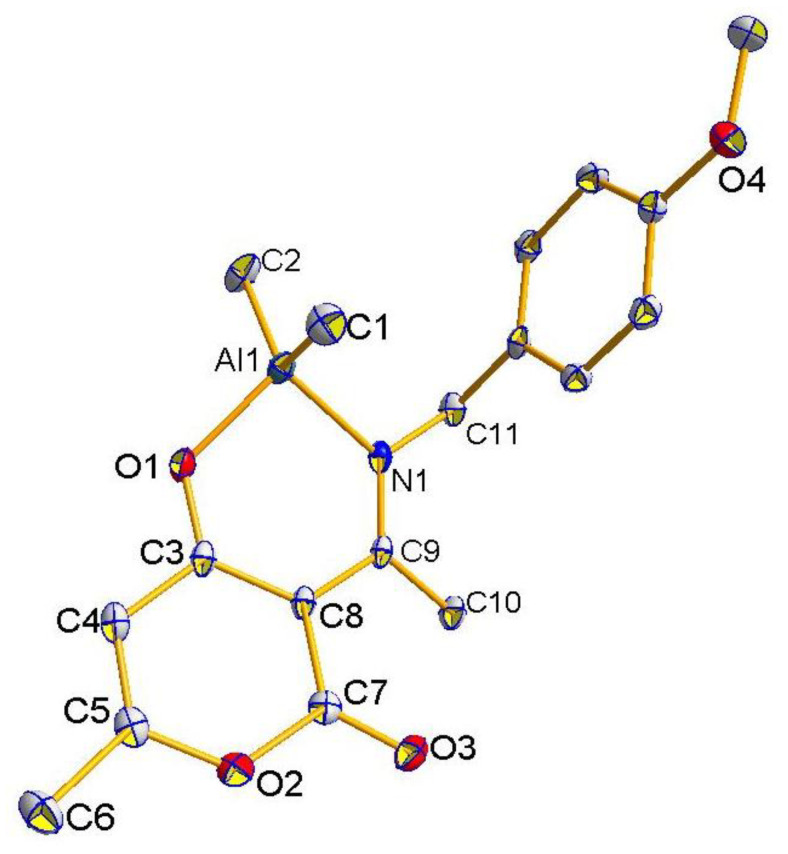
The molecular geometry of compound **3**. Thermal ellipsoids are drawn at the 30% probability level. THF and all the hydrogen atoms are omitted for clarity.

**Figure 4:**
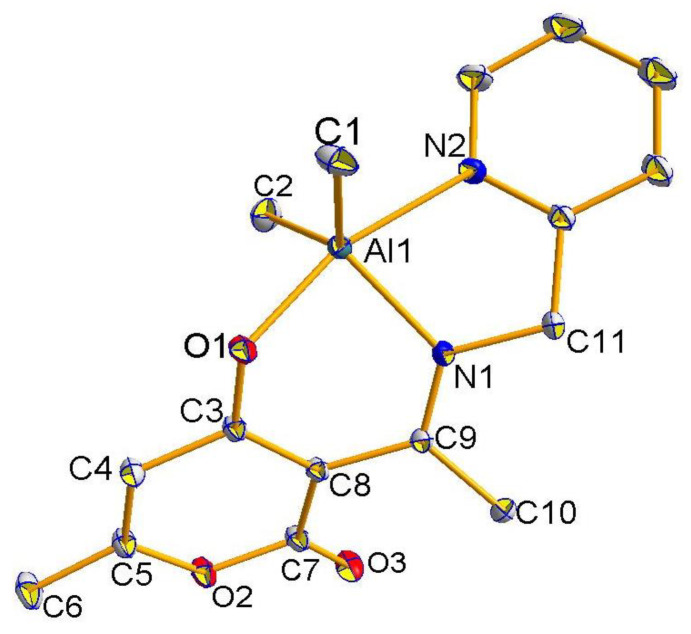
The molecular geometry of compound **4**. Thermal ellipsoids are drawn at the 30% probability level. THF and all the hydrogen atoms are omitted for clarity.

The crystals of **1**, **3**, and **4** were grown from saturated THF solution at −20 °C; the data collection processes and selective bond lengths and angles are listed in Tables S1 and S2, respectively. The molecular structures of **1**, **3**, and **4** are depicted in Figure 2, Figure 3 and Figure 4. The single-crystal X-ray structure reveals that the central Al atom of compounds **1** and **3** is tetragonally surrounded by the corresponding bidentate N, O-dehydroacetic acid–imine ligands and two methyl groups; thus, it exhibits a slightly distorted tetrahedral geometry. The biting angles for the bidentate N, O-dehydroacetic acid–imine ligands with aluminum atoms of compounds **1** and **3** are at 91.63(5) and 92.88(7)°, respectively. The bond lengths of Al-C_Me_, Al-N, and Al-O for compounds **1** and **3** are relatively similar, at ca. 1.95, 1.94, and 1.80 Å, respectively.

**Scheme 5:**
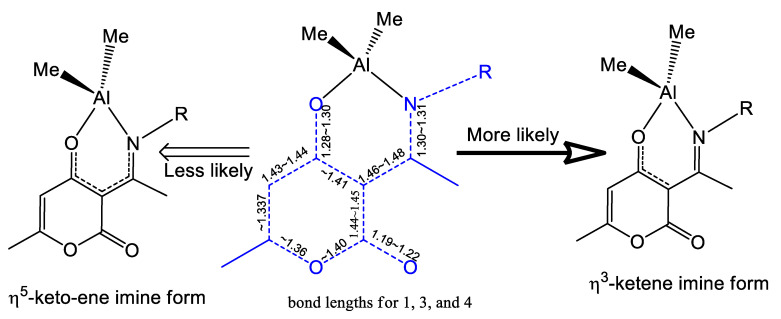
A summarization of bond lengths for **1**, **3**, and **4**, and their possible resonance forms.

The unit cell of compound **4** contains one Al-assisted molecular unit and two THF molecules. The molecular geometry of **4** possesses a distorted trigonal bipyramidal environment, where the dehydroacetic acid–imine ligand, **L^4^**, coordinates to the aluminum atom with the O(1) and N(2) at axial positions and the bond angle of O(1)-Al(1)-N(2) is at 163.45(5)°. The N(1) and two methyl carbon atoms occupy the equatorial positions, forming a trigonal plane where the total bond angle for C(1)-Al(1)-N(1), C(2)-Al(1)-N(1), and C(1)-Al(1)-C(2) is 359.3°. The bond lengths of Al(1)-Me, Al(1)-N(1), and Al(1)-O(1) for compound **4** are at ca. 1.99, 1.99, and 1.90 Å, respectively, which are slightly longer than those of the corresponding bond lengths of compounds **1** and **3**. Presumably, the pyridine coordinated aluminum atom of **4** enriches the electron density surrounding the metal and results in a lower electron withdrawing capacity from the surrounded methyl and dehydroacetic acid–imine ligands. The dehydroacetic acid–imine ligands **L^1^**, **L^3^**, and **L^4^** coordinate to the Al atom via N, O donor atoms to form the six-membered chelate ring. The O(1)-C(3) and C(3)-C(8) bond lengths for compounds **1**, **3**, and **4** are longer than their corresponding double bond disposition, which can be considered to possess a resonance ketene character. The bond lengths of C(8)-C(9) and C(9)-N(1) are in good agreement with the typical C-C single bond and C-N double bond characteristics. Therefore, the dehydroacetic acid–imine ligands **L^1^**, **L^3^**, and **L^4^** in compounds **1**, **3**, **4** can represent the η^3^-ketene imine conformation, as shown in Scheme 5. Similar bonding modes have been reported in the previous literature [38,39,40,41].

### 2.3. The Structure Resonance Forms of Ligands and Complexes in Solution

The solid-state structures of **L^1^H**~**L^4^H** evidence the ketone–imine arrangement, whereas the molecular structures of **1**, **3**, and **4** exhibit a ketene–imine conformation. Regarding the resonance forms for ligands **L^1^H**~**L^4^H**, as shown in Scheme 4, the structures in solid state are dominated by the imine ketone form, in accordance with the corresponding bond lengths. However, the resonance form may shift in solution states. The enamine ketone and enol imine forms were expected to possess a *sp*^2^ methine proton on the pyrone ring, and the imine ketone form should have one *sp*^2^ and one *sp*^3^ methine proton on the pyrone ring. The ^1^H-^13^C 2D HSQC NMR spectra of **L^1^H**~**L^4^H** show only the *sp*^2^ methine carbon and the proton cross peak; no *sp*^3^ methine proton peak was apprehended. Presumably, the ligands **L^1^H**~**L^4^H** in solution are influenced by the enamine ketone and enol imine dispositions.

The solid-state geometries of **1**, **3**, **4** are denoted as having an η^3^-ketene imine form, in accordance with their corresponding bond lengths, as shown in Scheme 5.

**Scheme 6:**
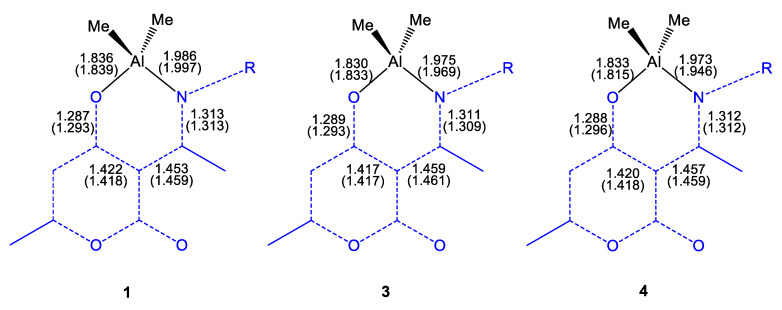
The calculated bond lengths of aluminum-N, O five-member rings of compounds **1**, **3**, and **4** in solid state and in THF (in parentheses) using B3LYP method.

However, η^5^-keto-ene imine, ketonene amide, and keto imine forms are other probable resonance forms that may exist in the metal complexes. The ^1^H-^13^C 2D-NMR spectra of complexes **1**~**4** do not conclude the differentiation of these conformations. Therefore, we performed the DFT B3LYP theoretical calculation [42,43] to determine the possible binding modes of compounds **1**~**4** in solid state as well as in THF solution. The bond-length comparisons of the theoretical calculations in solid state and in THF for compounds **1**~**4** are elucidated in Scheme 6. The theoretical data suggests that the aluminum atom binds to dehydroacetic acid–imine ligand more prominently, with a η^3^-ketene imine form in solid state and in THF for compounds **1**~**4**.

### 2.4. Catalytic Ring-Opening Polymerization of ε-Caprolactone and Coupling Reactions of Styrene Oxide with CO_2_

**Scheme 7:**
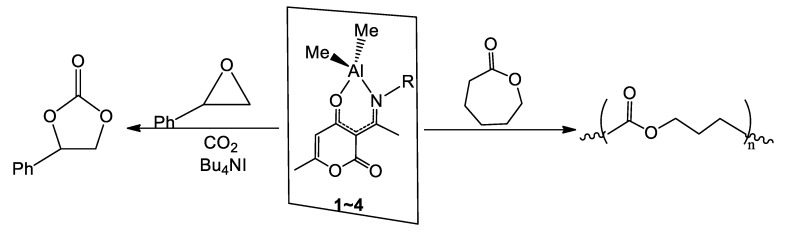
Aluminum compounds **1**–**4** as catalysts for CO_2_–epoxide coupling reactions and ring-opening polymerization of ε-caprolactone.

Compounds **1**–**4** were employed as initiators for the ring-opening polymerization of ε-caprolactone and as catalysts for the coupling reactions of styrene oxide with CO_2_, as shown in Scheme 7. Regarding the polymerization of ε-caprolactone, first, we selected compound **4** as the initiator for optimizing the solvent, reaction time, and temperature; the results are shown in Table 1.

Entries 1–3 show that the polymerization reaction of ε-caprolactone in toluene at 30 °C has an effective and controlled conversion rate in comparison with THF and DCM. Therefore, toluene was selected as an appropriate solvent for exploring the ROP catalysts. All the conversions were determined on the basis of integration of methylene protons at δ 4.24 ppm and 4.06 ppm from the ^1^H NMR spectra, as depicted in Figure S5. The poly ε-caprolactones were obtained by adding hexane to the reaction medium. The polymers were analyzed on the basis of ^1^H NMR spectra and gel permeation chromatography (GPC). As shown in entry 10, Table 1, the optimal conditions for the ring-opening polymerization of ε-caprolactone are the simultaneous addition of toluene as solvent and benzyl alcohol (BnOH) as co-catalyst at 80 °C for 1 h. These results are in agreement with reports in both the existing literature [44,45] and our previously published papers [46,47] that metal alkoxides are better initiators then metal alkyls for the ring-opening polymerization of cyclic esters. According to the practical data given in Table 2, compounds **1**~**4** all manifest promising activities for the ring-opening polymerization of ε-caprolactone at 80 °C and 1 h reaction time, with a conversion rate of ca. 99%. The PDI values for poly ε-caprolactone put through compounds **1**–**4** as initiators vary from 1.14 to 2.10. Two major factors might be accountable for broadening the PDI parameters: (i) possible intra- and inter-molecular transfer esterification; and (ii) the near homogeneity of the solution due to the large equivalents of monomer and the small amount of reacting solvent. These data are comparable to previous results [48], where Zn complexes were implemented as efficient initiators for the ROP of lactide, and the heterogeneous systems appeared to perform PLA with higher *M*_n_.

Compounds **1**~**4** were implemented as catalysts for the synthesis of cyclic styrene carbonates via the coupling reaction of styrene oxide and CO_2_ by varying the reaction time and using TBAI as co-catalyst (Scheme 7) [49,50,51,52]. The CO_2_ pressure was set at a little bit higher than 1 atm by using CO_2_ balloons, and reaction temperatures were set at 90 °C. The conversions of styrene oxides to styrene carbonates were determined by ^1^H NMR spectra to calculate the corresponding proton signal ratio of H_c_ vs. (H_f_ + H_c_), as shown in Figure S6. The impact of the catalysts coupled with the ligands, temperature and reaction time was inspected during the coupling reaction. The results outlined in Table 3 reveal that each of the compounds **1**–**4** possesses efficient catalytic activity towards styrene oxide and CO_2_ coupling reaction with long time durations, thus achieving over 90% conversion after 12 h at 90 °C. This result is comparable with a recent study [53] in which the tetranuclear Zn(II) complex with polyhedral oligomeric silsesquioxane was used as a catalyst for the synthesis of cyclic carbonates from epoxide and CO_2_. High yields of cyclic carbonates were achieved for terminal epoxides under mild conditions. By comparing the conversions of the CO_2_/styrene oxide coupling reaction using catalysts **1**–**4** at 90 °C for 6 h, the catalytic activity was in the order **1** > **4** > **3** > **2**. These results indicate that the steric hindrance of these complexes does not play an important role in the catalytic activity. The electronic effects have important impacts on the catalytic activity of catalysts **1**–**4**. In addition, entries 1, 4, 7 and 10 of Table 3 show low conversions of CO_2_/styrene oxide coupling reaction at 90 °C for 3 h, indicating that the catalytic reactions required an induction period for all of the catalysts [54].

## 3. Conclusions

In conclusion, we synthesized a series of dehydroacetic acid–imine ligands **L^1^H**~**L^4^H** and their corresponding aluminum compounds **1**–**4**. All the ligands and aluminum derivatives were characterized by means of ^1^H and ^13^C{^1^H} NMR spectra along with single-crystal X-ray diffractometry. The six-membered ring Al derivatives, **1**–**4**, were distinctly beneficial, with competent propagation activity towards the ROP of ε-caprolactones as well as predominant towards the coupling reactions of styrene oxides with CO_2_. In future research, further optimization of the polymerization kinetics, more controlled effect of the new catalysts, and mechanistic study of epoxide/CO_2_ copolymerization will be proposed and investigated.

## 4. Experimental Section

### 4.1. Physical Experiments and Reagents

All the reactions were performed using standard Schlenk techniques in nitrogen atmosphere or in glove box. Toluene and diethyl ether were dried over Na/benzophenone ketyl and distilled prior to use. CH_2_Cl_2_ was dried over P_2_O_5_ and distilled. **L^1^H**~**L^4^H** were synthesized according to the published literature. ^11^ CDCl_3_ was degassed using freeze-and-thaw cycles and dried over 4Å molecular sieves. ^1^H and ^13^C NMR spectra were recorded on a Bruker Avance 300 spectrometer. Chemical shifts for ^1^H and ^13^C{1H} spectra were recorded in ppm relative to the residual protons and ^13^C{1H} of CDCl_3_ (δ 7.24, 77.0 ppm) and C_6_D_6_ (δ 7.15, 128.0 ppm). Elemental analyses were performed on a Heraeus CHN-OS Rapid Elemental Analyzer at the Instrument Center, NCHU. Due to high moisture sensitivity and rapid decomposition, the elemental analyses of some metal complexes were not in the error range.

### 4.2. Synthesis of Ligands ***L^1^H**~**L^4^H*** and Complexes ***1**~**4***

Synthesis of **L^1^H**. A 250 mL round bottom flask was charged with 3.00 g (17.8 mmol) of dehydroacetic acid, 2.65 g of 2-*^t^*butylaniline (17.8 mmol), and 150 mL of methanol. A few drops of formic acid were added as catalyst. The solution was refluxed for 16 h and then the volatiles were removed under vacuum. The resulting solid was recrystallized with heptane to give white crystals of **L^1^H** in 74% (4.18 g). ^1^H NMR (δ, CDCl_3_): 7.48 (m, 1H, phenyl C*H*), 7.29 (m, 2H, phenyl C*H*), 6.93 (s, 1H, phenyl C*H*), 5.75 (s, 1H, =C*H*), 2.45 (s, 3H, *Me*), 2.13 (s, 3H, *Me*), 1.32 (s, 9H, *Me*). ^13^C NMR (δ, CDCl_3_): 184.9, 175.9, 163.5, 145.6, 135.0, 128.9, 128.7, 127.6, 127.1, 107.2, 97.3, 58.3 (solvent DCM), 35.0, 30.6, 21.1, 20.0, 18.4. Anal. Cacld. for C_18_H_21_NO_3_ (299.36): C, 72.22; H, 7.07; N, 4.68. Found: C, 71.15; H, 7.57; N, 4.42%. m.p. (melting point): 107 °C. MS (EI, M+): 299.

Synthesis of **L^2^H**. A similar procedure to that for synthesizing **L^1^H** was adopted. Dehydroacetic acid (3.00 g, 17.8 mmol), (S)-(-)-1-phenylethylamine (2.15 g, 17.8 mmol), and 150 mL of methanol were used and 4.86 g of solids was obtained (80%). ^1^H NMR (δ, CDCl_3_): 7.31 (m, 5H, phenyl C*H*), 5.68 (s, 1H, =C*H*), 4.96 (q, 1H, C*H*), 2.51 (s, 3H, *Me*), 2.10 (d, ^4^*J*_HH_ = 2 Hz, 3H, *Me*), 1.65 (d, ^3^*J*_HH_ = 7 Hz, 3H, *Me*). ^13^C NMR (δ, CDCl_3_): 184.8, 175.5, 163.7, 162.7, 141.6, 129.2, 127.9, 125.6, 107.3, 96.7, 54.4, 24.0, 19.7, 18.7. Anal. Cacld. for C_16_H_17_NO_3_ (271.31): C, 70.83; H, 6.32; N, 5.16. Found: C, 70.80; H, 6.59; N, 5.12%. m.p. (melting point): 130 °C. MS (EI, M+): 271.

Synthesis of **L^3^H**. A similar procedure to that for synthesizing **L^1^H** was adopted. Dehydroacetic acid (3.00 g, 17.8 mmol), 4-methoxybenzylamine (2.44 g, 17.8 mmol), and 150 mL of methanol were used and 4.26 g of solids was obtained (83%). ^1^H NMR (δ, CDCl_3_): 7.17 (m, 2H, phenyl C*H*), 6.86 (m, 2H, phenyl C*H*), 5.63 (d, ^4^*J*_HH_ = 2 Hz, 1H, =C*H*), 4.56 (d, 2H, C*H*_2_), 3.75 (s, 3H, O*Me*), 2.63 (s, 3H, *Me*), 2.07 (d, ^4^*J*_HH_ = 2 Hz, 3H, *Me*). ^13^C NMR (δ, CDCl_3_): 184.6, 176.0, 163.7, 162.7, 159.4, 129.6, 126.9, 114.5, 107.3, 96.7, 55.2, 47.4, 19.7, 18.3. Anal. Cacld. for C_16_H_17_NO_4_ (287.31): C, 66.89; H, 5.96; N, 4.88. Found: C, 66.95; H, 5.90; N, 4.78%. m.p. (melting point): 109 °C. MS (EI, M+): 287.

Synthesis of **L^4^H**. A similar procedure to that for synthesizing **L^1^H** was adopted. Dehydroacetic acid (3.00 g, 17.8 mmol), 2-(aminomethyl)pyridine (1.92 g, 17.8 mmol), and 150 mL of methanol were used and 2.02 g of solids was obtained (47%). ^1^H NMR (δ, CDCl_3_): 8.59 (m, 1H, pyridine C*H*), 7.67 (m, 1H, pyridine C*H*), 7.22 (m, 2H, pyridine C*H*), 5.65 (s, 1H, =C*H*), 4.78 (d, 2H, C*H*_2_), 2.66 (s, 3H, *Me*), 2.07 (s, 3H, *Me*). ^13^C NMR (δ, CDCl_3_): 184.6, 176.4, 163.7, 162.7, 154.3, 149.8, 137.1, 122.9, 121.3, 107.3, 96.9, 49.2, 19.7, 18.6. Anal. Cacld. for C_14_H_14_N_2_O_3_ (258.27): C, 65.11; H, 5.46; N, 10.85. Found: C, 65.07; H, 5.36; N, 10.85%. m.p. (melting point): 118 °C. MS (EI, M+): 258.

Synthesis of compound **AlMe_2_L^1^** (**1**). To a toluene (15 mL) solution of compound **L^1^H** (1.00 g, 2.8 mmol) in a Schlenk flask, AlMe_3_ was added in toluene (2M in toluene, 1.40 mL, 2.8 mmol) dropwise at 0 °C. The solution was stirred at room temperature for 3 h and volatiles were removed under vacuum to yield orange solid. The product was recrystallized from a saturated THF solution at −20 °C to give 0.86 g of pale orange crystals (86% yield). ^1^H NMR (C_6_D_6_): 7.23 (m, 1H, phenyl C*H*), 6.89 (m, 2H, phenyl C*H*), 6.58 (m, 1H, phenyl C*H*), 5.43 (s, 1H, =C*H*), 2.35 (s, 3H, *Me*), 1.38 (s, 3H, *Me*), 1.19 (s, 9H, *Me*), −0.36 (s, 3H, *Me*), −0.40 (s, 3H, *Me*). ^13^C NMR (C_6_D_6_): 179.6, 179.5, 166.8, 162.3, 142.3, 140.7, 131.1, 128.3, 127.5, 126.1, 105.4, 101.3, 36.4, 32.7, 24.3, 19.3, −10.0. Due to air sensitivity of the complex, the error range of the elemental analysis data was a little bit higher than expected.

Synthesis of compound **AlMe_2_L^2^** (**2**). Similar procedure to that for synthesizing **AlMe_2_L^1^** (**1**) was adopted. Ligand **L^2^H** (1.00 g, 3.0 mmol), AlMe_3_ (2M in toluene, 1.50 mL, 3.0 mmol), and toluene (15 mL) were used. The solution was stirred at room temperature for 3 h and volatiles were removed under vacuum to give pale yellow sticky liquid 0.61 g, yield 62%. ^1^H NMR (C_6_D_6_): 7.05 (m, 5H, phenyl C*H*), 5.47 (s, 1H, =C*H*), 4.59 (q, 1H, C*H*), 2.24 (s, 3H, *Me*), 1.43 (s, 3H, *Me*), 1.41 (s, 3H, *Me*), −0.39 (s, 3H, *Me*), −0.59 (s, 3H, *Me*). ^13^C NMR (C_6_D_6_): 178.2, 178.1, 166.0, 162.4, 139.9, 128.9, 127.7, 127.1, 105.0, 102.5, 58.5, 21.5, 20.0, 19.2, −8.5. Due to air sensitivity of the complex, the error range of the elemental analysis data was a little bit higher than expected.

Synthesis of compound **AlMe_2_L^3^** (**3**). Similar procedure to that for synthesizing **AlMe_2_L^1^** (**1**) was adopted. Ligand **L^3^H** (1.00 g, 2.9 mmol), AlMe_3_ (2M in toluene, 1.50 mL, 3.0 mmol), and toluene (15 mL) were used. The solution was stirred at room temperature for 3 h and volatiles were removed under vacuum to give 0.67 g of yellowish solid (65% yield). ^1^H NMR (C_6_D_6_): 6.82 (m, 2H, phenyl C*H*), 6.67 (m, 2H, phenyl C*H*), 5.44 (d, ^4^*J*_HH_ = 2 Hz, 1H, =C*H*), 4.22 (s, 2H, C*H*_2_), 3.25 (s, 3H, O*Me*), 2.25 (s, 3H, *Me*), 1.39 (d, ^4^*J*_HH_ = 2 Hz, 3H, *Me*), −0.39 (s, 6H, *Me*). ^13^C NMR (C_6_D_6_): 179.9, 178.3, 166.0, 162.4, 159.6, 128.5, 125.6, 114.5, 105.3, 101.9, 54.8, 51.6, 21.2, 19.3, −10.3. Anal. Cacld. for C_18_H_22_AlNO_4_ (343.35): C, 62.97; H, 6.46; N, 4.08. Found: C, 62.93; H, 6.32; N, 4.00%.

Synthesis of compound **AlMe_2_L^4^** (**4**). Similar procedure to that for synthesizing **AlMe_2_L^1^** (**1**) was adopted. Ligand **L^4^H** (1.00 g, 2.8 mmol), AlMe_3_ (2M in toluene, 1.40 mL, 2.80 mmol), and toluene (15 mL) were used. The solution was stirred at room temperature for 3 h and volatiles were removed under vacuum to give pale pink solid. The solid was recrystallized from a saturated THF solution at −20 °C to give pink crystals 0.68 g (73% yield). ^1^H NMR (C_6_D_6_): 8.18 (m, 1H, pyridine C*H*), 6.92 (m, 1H, pyridine C*H*), 6.54 (m, 1H, pyridine C*H*), 6.27 (m, 1H, pyridine C*H*), 5.69 (d, ^4^*J*_HH_ = 2 Hz, 1H, =C*H*), 3.86 (s, 2H, C*H*_2_), 3.56 (m, *THF*), 2.20 (s, 3H, *Me*), 1.45 (s, 3H, *Me*), 1.43 (m, *THF*), −0.20 (s, 6H, *Me*). ^13^C NMR (C_6_D_6_): 179.2, 179.0, 164.5, 163.4, 153.8, 144.9, 138.1, 123.2, 121.1, 106.0, 101.5, 67.8, 52.3, 25.8, 22.6, 19.4, −5.8. Anal. Cacld. for C_16_H_19_AlN_2_O_3_ (314.32): C, 61.14; H, 6.09; N, 8.91. Found: C, 60.89; H, 6.06; N, 8.57%.

### 4.3. Ring-Opening Polymerization of ε-Caprolactone

In a typical procedure, the initiator (0.05 mmol) was first dissolved in 2.5 mL of solvent followed by the addition of ε-caprolactone and then stirred at specific temperature and time to produce a gel- or solid-like polymer. The mixture was quenched with distilled water, and the resulting solid was washed with hexane and methanol. It was dried and gave a satisfactory yield. The molecular weight of the polymers was determined using a gel permeation chromatography (GPC) instrument (Waters, RI 2414, pump 1515).

### 4.4. CO_2_ Coupling Reactions with Styrene Oxide

The general procedure for the coupling reaction is shown below. A Schlenk flask was charged with 0.02 mmol of catalyst and 50 equiv. of styrene oxide. TBAI (1equiv) and CO_2_ balloon were used. The reaction temperature was set at 90 °C and the conversion was determined by ^1^H NMR spectra.

### 4.5. X-ray Crystallography

Suitable crystals of **L^1^H**~**L^4^H** and compounds **1**, **3** and **4** were attached to a fine glass fiber and mounted on goniostat for structural refinement. Data collection was performed at 150 K under liquid nitrogen vapor for all compounds. Data were collected on a Bruker SMART CCD diffractometer with graphite monochromated Mo-K_α_ radiation. No significant crystal decay was found. Data were corrected for absorption empirically by means of *ψ* scans. All non-hydrogen atoms were refined with anisotropic displacement parameters. For all the structures, the hydrogen atom positions were calculated, and they were constrained to idealized geometries and treated as riding, where the H atom displacement parameter was calculated from the equivalent isotropic displacement parameter of the bound atom. An absorption correction was performed with the program SADABS [55] and the structures of both complexes were determined by direct methods procedures in SHELXS [56] and refined by full-matrix least-squares methods, on *F*^2^’s, in SHELXL [57]. All the relevant crystallographic data and structure refinement parameters are summarized in Table S1.

## Figures and Tables

**Table 1:** Ring-opening polymerization of ε-CL using compound **4** as initiator ^a^ in different solvents.

Entry	Solvent	Time (h)	Temp (C)	BnOH ^b^	Conv. ^c^ (%)
1	THF	24	30	-	7.4
2	DCM	24	30	-	3.8
3	toluene	24	30	-	12.6
4	toluene	1	30	-	NR
5	toluene	1	80	-	15
6	toluene	3	80	-	47
7	toluene	6	80	-	56
8	toluene	1	30	1	NR
9	toluene	1	60	1	66
10	toluene	1	80	1	99

^a^ Reaction condition: 0.05 mmol of catalyst; [M]/[I] = 50; solvent = 2.5 mL. ^b^ [BnOH] = 0.1 M. ^c^ Monomer conversion as determined by ^1^H NMR spectra.

**Table 2:** Ring-opening polymerization of ε-CL using compounds **1**–**4** as initiator ^a^.

Entry	Initiator	[M]/[I]	M_n, exp_. ^b^ (g/mol)	M_n, theo_. (g/mol)	M_w_	PDI	Conv. ^c^ (%)
1	**1**	50/1	5900	5700	12,000	1.14	99
2	**1**	100/1	14,800	11,300	45,600	1.51	99
3	**1**	150/1	16,900	16,900	44,300	1.67	99
4	**1**	200/1	27,900	22,600	78,700	1.56	99
5	**1**	250/1	28,200	25,700	66,800	1.34	90
6	**2**	50/1	6200	5700	13,100	1.19	99
7	**2**	100/1	9700	10,800	22,200	1.28	95
8	**2**	150/1	6600	14,700	21,300	1.79	86
9	**2**	200/1	27,200	22,600	75,000	1.54	99
10	**2**	250/1	26,100	28,000	98,000	2.10	99
11	**3**	50/1	7600	5700	16,400	1.20	99
12	**3**	100/1	10,800	11,300	31,800	1.65	99
13	**3**	150/1	15,200	16,900	48,900	1.80	99
14	**3**	200/1	17,200	21,700	44,700	1.39	95
15	**3**	250/1	17,900	28,300	47,300	1.54	99
16	**4**	50/1	7500	5700	20,000	1.48	98
17	**4**	100/1	8000	11,000	19,900	1.38	97
18	**4**	150/1	13,000	11,300	33,900	1.46	99
19	**4**	200/1	14,800	16,900	39,500	1.48	95
20	**4**	250/1	21,500	28,300	69,200	1.75	99

^a^ Reaction conditions: 0.05 mmol of catalyst, solvent = 2.5 mL, [BnOH] = 0.1 M, 80 °C, 1 h, ^b^ Obtained from GPC analysis using a column calibrated by polystyrene standard, multiplied by a correcting factor of 0.56. ^c^ Monomer conversion as determined by ^1^H NMR spectra.

**Table 3:** Styrene oxide and CO_2_ coupling reactions using compounds **1**~**4** as catalysts ^a^.

Entry	Catalyst	Temp. (°C)	Time (h)	Conv.% ^b^
1	1	90	3	2.9
2	1	90	6	42
3	1	90	12	85
4	2	90	3	4.7
5	2	90	6	11
6	2	90	12	91
7	3	90	3	2.8
8	3	90	6	23
9	3	90	12	95
10	4	90	3	5.9
11	4	90	6	31
12	4	90	12	93

^a^ Reaction conditions: [cat.]/[styrene oxide] = 1/50, CO_2_ 1 bar, reaction temperature 90 °C, TBAI = 1 eq. TBAI. ^b^ Conversion determined by ^1^H NMR spectra.

## Data Availability

Not applicable.

## Supplementary Materials

The following are available online, Figure S1: The molecular geometry of ligand L1H. Thermal ellipsoids are drawn at 30% probability level, Figure S2: The molecular geometry of ligand L2H. Thermal ellipsoids are drawn at 30% probability level. Figure S3: The molecular geometry of ligand L3H. Thermal ellipsoids are drawn at 30% probability level. Figure S4: The molecular geometry of ligand L4H. Thermal ellipsoids are drawn at 30% probability level. Figure S5: 1H NMR spectra of (a) ε-caprolactone (b) poly-ε-caprolactone in CDCl3 using 300 MHz NMR spectrometer. Figure S6: The 1H NMR spectra showing the proton signals of (A) styrene oxide and (B) mixture of styrene oxide and styrene carbonate in the range of δ 6.0~2.0. Table S1: The summary of X-ray crystal data for L1H~L4H, 1, 3, and 4, Table S2: Selected bond lengths and angles for L1H~L4H, 1, 3, and 4, Crystallographic data for the structures reported in this paper have been deposited with the Cambridge Crystallographic Data Centre as supplementary publications nos. CCDC-~2070248-2070253 (compounds **L^1^H**~**L^4^H** and compounds **1**, **3** and **4**). Copies of the data can be obtained free of charge on application to CCDC, 12 Union Road, Cambridge CB2 1EZ, UK (Fax: +44-1223-336-033; e-mail: deposit@ccdc.cam.ac.uk or http://www.ccdc.cam.ac.uk, accessed on 23 September 2021).

## Sample Availability

Samples of the compounds are not available from the authors.
