# Supplementary material for: Ring-Opening Polymerization of ε-Caprolactone and Styrene Oxide–CO2 Coupling Reactions Catalyzed by Chelated Dehydroacetic Acid–Imine Aluminum Complexes
Source: Molecules. 2021 Dec 28;27(1):164. doi: 10.3390/molecules27010164 (PMC8746973; doi:10.3390/molecules27010164)
Supplement: Supplementary file 1 [file molecules-27-00164-s001.zip › molecules-1500611-supplementary.pdf]

molecules

MDPI

Electronic Supplementary Information

Ring Opening Polymerization of ε-Caprolactone and Styrene Oxide-CO_2 Coupling Reactions Catalyzed by Chelated Dehydroacetic Acid-imine Aluminum Complexes

Ting-Yen Wang [1], Yu-Chia Su [2], Bao-Tsan Ko [2], Yu Hsu[1], Yu-Fang Zeng [1], Ching-Han Hu [1], Amitabha Datta [1] and Jui-Hsien Huang [1,*]

Table S1. The summary of X-ray crystal data for **L_1H~L_4H**, **1**, **3**, and **4**

	**L_1H**	**L_2H**	**L_3H**	**L_4H**	**1**	**3**	4·2THF
formula	$C_{18}H_{21}NO_3$	$C_{16}H_{17}NO_3$	$C_{16}H_{17}NO_4$	$C_{14}H_{14}N_2O_3$	$C_{20}H_{26}AlNO_3$	$C_{18}H_{22}AlNO_4$	$C_{24}H_{35}AlN_2O_5$
FW	299.36	271.30	287.30	258.27	355.40	343.34	458.52
T [K]	150	150	150	150	150	296	150
crystal system	Orthorhomb ic	Orthorhomb ic	Monoclinic	Monoclinic	Monoclinic	Orthorhomb ic	Monoclinic
space group	Pbcn	$P2_12_12_1$	P1 21/c 1	C2/c	$P2_1/n$	Pbcn	$P2_1/c$
a [Å]	10.7230(4)	10.7376(6)	3.9467(9)	30.7175(9)	10.9949(5)	34.8423(13)	13.8302(19)
b [Å]	15.2288(6)	11.2814(4)	27.567(6)	17.7730(6)	12.7574(5)	7.4216(3)	14.304(2)
c [Å]	21.0174(9)	11.6410(6)	12.564(3)	13.7716(5)	13.9045(5)	13.6698(5	13.8016(17)
α [º]	90	90	90	90	90	90	90
β [º]	90	90	92.389(8)	90.701(2)	98.891(2)	90	112.082(6)
γ [º]	90	90	90	90	90	90	90
V [Å^3]	3432.1(2)	1410.13(12)	1365.8(5)	7517.9(4)	1926.90(14)	3534.8(2)	2530.0(6)
Z	8	4	4	24	4	8	4
ρ_c [Mg m^{-3}]	1.159	1.278	1.397	1.369	1.225	1.290	1.204

μ[mm^{-1}]	0.079	0.088	0.101	0.098	0.123	0.136	0.115
F(000)	1280	576	608	3264	760	1456	984
rflns collected	59750	26689	19454	62025	35324	62164	35563
independent rflns	3015 [R_{int}=0.0537]	3337 [R_{int}=0.0382]	2399 [R_{int}=0.0913]	6583 [R_{int}=0.0415]	4572 [R_{int}=0.0395]	4561 [R_{int}=0.0422]	6566 [R_{int}=0.0307]
data/restraints/ parameters	3015/0/204	3337/0/184	2399/0/194	6583/0/520	4572/0/233	4561/0/222	6566/0/293
goodness-of-fit on F^2	1.059	1.133	0.781	1.122	1.071	1.509	1.098
R_1, wR_2 (I>2σ(I))	R_1=0.0366, wR_2=0.0949	R_1=0.0360, wR_2=0.1004	R_1=0.0781, wR_2=0.2288	R_1=0.0552, wR_2=0.1625	R_1=0.0426, wR_2=0.1257	R_1=0.0526, wR_2=0.1870	R_1=0.0484, wR_2=0.1548
R_1, wR_2 (all data)	R_1=0.04626, wR_2=0.1033	R_1=0.0412, wR_2=0.11139	R_1=0.1021, wR_2=0.2603	R_1=0.0679, wR_2=0.1787	R_1=0.0518, wR_2=0.1398	R_1=0.0652, wR_2=0.1956	R_1=0.0560, wR_2=0.1624
largest diff. peak, hole [*e*Å^{-3}]	0.194/-0.187	0.285/-0.306	0.707/-0.515	0.394/-0.364	0.602/-0.372	0.278/-0.685	0.532/-0.589

Table S2. Selected bond lengths and angles for **L_1H~L_4H**, **1**, **3**, and **4**

**L_1H**			
N(1)-C(7)	1.3162(18)	O(2)-C(3)	1.3661(18)
O(2)-C(5)	1.3947(17)	O(3)-C(5)	1.2160(18)
O(1)-C(1)	1.2586(18)	C(7)-C(6)	1.429(2)
C(1)-C(2)	1.443(2)	C(3)-C(2)	1.331(2)
C(6)-C(1)	1.4448(19)	N(1)-C(9)	1.4385(18)
C(6)-C(5)	1.438(2)		
**L_2H**			
O(2)-C(3)	1.364(3)	O(2)-C(5)	1.403(2)
O(3)-C(5)	1.210(2)	O(1)-C(1)	1.258(2)
N(1)-C(7)	1.313(2)	C(7)-C(6)	1.433(2)
C(6)-C(5)	1.439(2)	C(6)-C(1)	1.442(2)
C(1)-C(2)	1.451(2)	N(1)-C(9)	1.474(2)

C(3)-C(2)	1.324(3)		
**L_3H**			
O(1)-C(1)	1.207(5)	C(2)-C(3)	1.329(5)
C(2)-C(5)	1.450(5)	O(3)-C(5)	1.253(4)
N(1)-C(7)	1.318(5)	C(1)-O(2)	1.398(5)
C(6)-C(7)	1.422(5)	C(5)-C(6)	1.441(5)
C(1)-C(6)	1.442(5)	N(1)-C(9)	1.471(5)
O(2)-C(3)	1.355(5)		
**L_4H**			
O(1)-C(10)	1.246(3)	O(2)-C(12)	1.365(3)
O(2)-C(14)	1.402(3)	N(2)-C(6)	1.456(3)
C(9)-C(7)	1.432(3)	C(9)-C(14)	1.434(3)
C(11)-C(12)	1.338(4)	C(10)-C(11)	1.438(4)
C(7)-C(8)	1.500(3)	C(9)-C(10)	1.442(3)
N(2)-C(7)	1.308(3)	O(3)-C(14)	1.224(3)
**1**			
Al(1)-O(1)	1.8031(11)	Al(1)-C(1)	1.9576(15)
Al(1)-C(2)	1.9571(16)	Al(1)-N(1)	1.9455(12)
C(9)-C(8)	1.4636(19)	O(1)-C(3)	1.2962(17)
O(2)-C(5)	1.352(2)	C(5)-C(4)	1.336(2)
O(3)-C(7)	1.194(2)	O(2)-C(7)	1.399(2)
C(8)-C(7)	1.451(2)	C(8)-C(3)	1.410(2)
C(3)-C(4)	1.4351(19)	N(1)-C(9)	1.3066(17)
N(1)-C(11)	1.4479(16)		
O(1)-Al(1)-C(2)	108.45(6)	O(1)-Al(1)-N(1)	91.63(5)
O(1)-Al(1)-C(1)	111.60(6)	N(1)-Al(1)-C(2)	109.45(6)
C(2)-Al(1)-C(1)	120.34(7)	N(1)-Al(1)-C(1)	111.61(6)
**3**			
Al(1)-O(1)	1.7968(15)	Al(1)-N(1)	1.9436(15)
Al(1)-C(1)	1.949(2)	Al(1)-C(2)	1.954(2)
O(2)-C(5)	1.355(2)	O(2)-C(7)	1.399(2)
O(3)-C(7)	1.213(2)	O(1)-C(3)	1.302(2)
N(1)-C(9)	1.305(2)	N(1)-C(11)	1.478(2)
C(9)-C(8)	1.465(2)	C(8)-C(3)	1.408(2)
C(8)-C(7)	1.443(2)	C(5)-C(4)	1.337(3)
C(3)-C(4)	1.431(2)		
O(1)-Al(1)-N(1)	92.88(7)	O(1)-Al(1)-C(1)	109.01(8)
N(1)-Al(1)-C(1)	109.79(8)	O(1)-Al(1)-C(2)	109.54(9)

N(1)-Al(1)-C(2)	110.50(8)	C(1)-Al(1)-C(2)	121.38(10)
**4**			
Al(1)-O(1)	1.8984(10)	Al(1)-C(2)	1.9759(16)
Al(1)-C(1)	1.9834(15)	Al(1)-N(1)	1.9932(11)
Al(1)-N(2)	2.1218(12)	O(1)-C(3)	1.2824(15)
O(3)-C(7)	1.2151(16)	O(2)-C(5)	1.3643(16)
O(2)-C(7)	1.3997(15)	C(4)-C(5)	1.3372(18)
C(4)-C(3)	1.4394(17)	C(7)-C(8)	1.4379(17)
C(8)-C(3)	1.4115(16)	C(8)-C(9)	1.4570(16)
N(1)-C(9)	1.3042(16)		
O(1)-Al(1)-C(2)	96.94(6)	O(1)-Al(1)-C(1)	93.92(5)
C(2)-Al(1)-C(1)	124.49(7)	O(1)-Al(1)-N(1)	87.00(4)
C(2)-Al(1)-N(1)	116.08(6)	C(1)-Al(1)-N(1)	118.73(6)
O(1)-Al(1)-N(2)	163.45(5)	C(2)-Al(1)-N(2)	94.67(6)
C(1)-Al(1)-N(2)	89.16(6)	N(1)-Al(1)-N(2)	77.30(5)

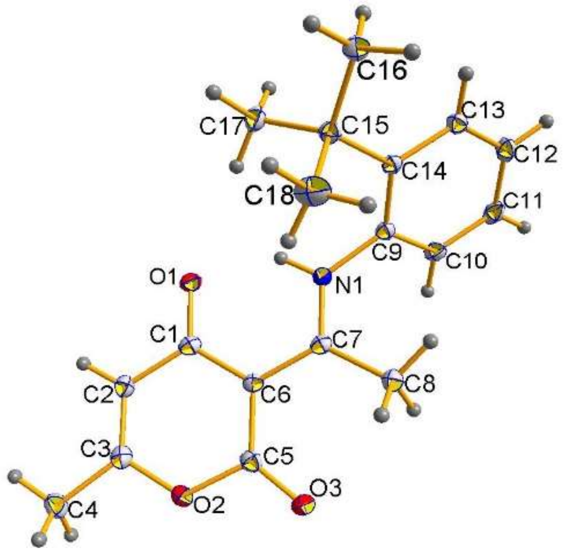

Figure S1. The molecular geometry of ligand **L¹H**. Thermal ellipsoids are drawn at 30% probability level.

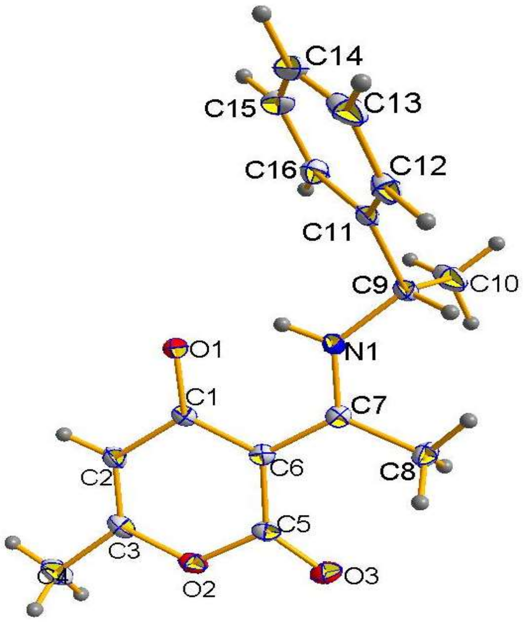

Figure S2. The molecular geometry of ligand **L²H**. Thermal ellipsoids are drawn at 30% probability level.

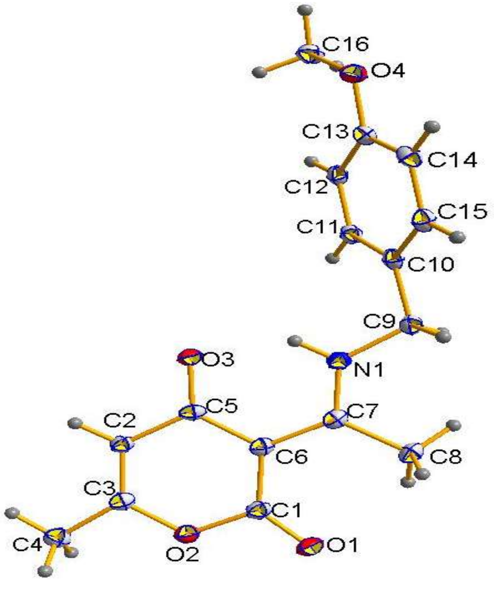

Figure S3. The molecular geometry of ligand **L³H**. Thermal ellipsoids are drawn at 30% probability level.

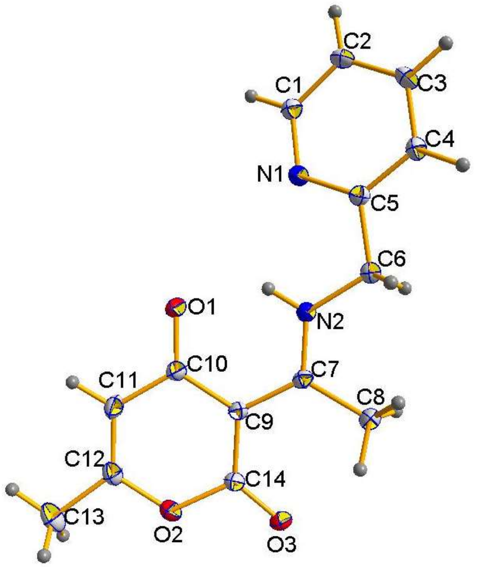

Figure S4. The molecular geometry of ligand **L⁴H**. Thermal ellipsoids are drawn at 30% probability level.

(a)

(b)

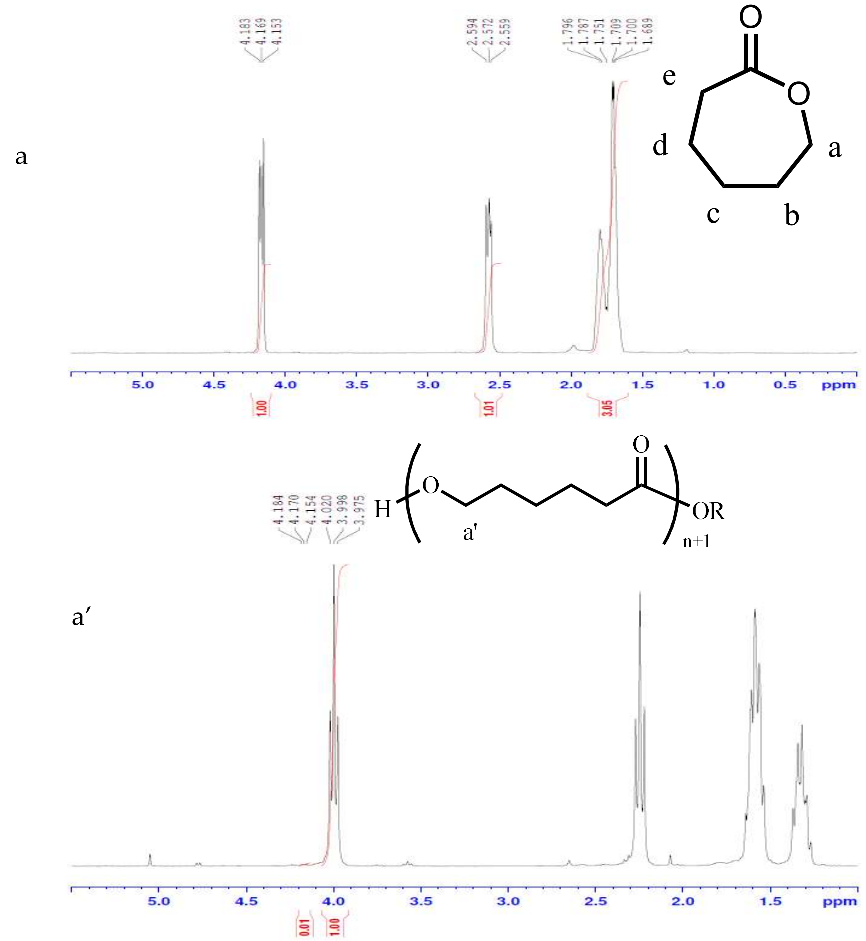

Figure S5. ^{1}H NMR spectra of (a) ε-caprolactone (b) poly-ε-caprolactone in $CDCl_3$ using 300 MHz NMR spectrometer.

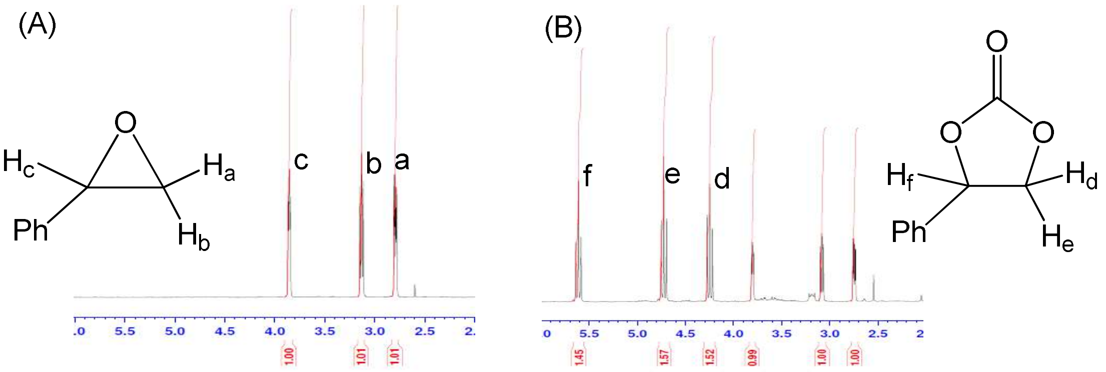

Figure S6. The ^{1}H NMR spectra showing the proton signals of (A) styrene oxide and (B) mixture of styrene oxide and styrene carbonate in the range of δ 6.0~2.0
